# Advantage of cerebellar intermittent theta-burst stimulation in Alzheimer's disease (ACT-AD): study protocol for a multicenter, randomized, double-blind, sham-controlled trial

**DOI:** 10.1186/s13063-026-10009-w

**Published:** 2026-10-09

**Authors:** Xin Zhang, Wen Li, Zhongqing Sun, Xiaomu Wang, Changgeng Song, Xiaorui Wang, Yinuo Niu, Dong Wei, Wei Zhang, Gejuan Zhang, Bei Zhang, Ling Wang, Zhirong Liu, Jingjing Zhao, Wen Jiang

**Affiliations:** 1 Department of Neurology, Xijing Hospital, Fourth Military Medical University, Xi’an, 710032 China; 2 Department of Neurology, Tangdu Hospital, Fourth Military Medical University, Xi’an, Shaanxi Province 710038 China; 3 Department of Neurology, Xi’an No.3 Hospital, The Affiliated Hospital of Northwest University, Xi’an, Shaanxi Province 710000 China; 4 Department of Neurology, The First Affiliated Hospital of Xi’an Medical University, Xi’an, 710077 China; 5 Department of Health Statistics, Fourth Military Medical University, Xi’an, 710032 China

**Keywords:** Alzheimer’s disease, Transcranial magnetic stimulation, Cerebellum, Theta burst stimulation

## Abstract

**Introduction:**

The cerebellum plays a crucial role in cognitive processing and thus presents a promising therapeutic target for Alzheimer’s disease (AD). This trial aims to evaluate the efficacy and safety of long-term, neuronavigated cerebellar intermittent theta burst stimulation (iTBS) in patients with AD.

**Methods and analysis:**

The ACT-AD trial is a multicenter, randomized, double-blind, sham-controlled study involving 100 participants with mild-to-moderate AD across 4 centers in China. Participants will be randomized to receive either cerebellar iTBS or sham stimulation for 4 weeks (daily sessions), followed by 8 weeks of weekly maintenance sessions. The primary outcome is the change in the Clinical Dementia Rating Scale Sum of Boxes (CDR-SB) score from baseline to week 12. Secondary outcomes include additional clinical scales. Exploratory outcomes include electroencephalogram (EEG), blood biomarkers and magnetic resonance imaging (MRI). Statistical analysis will follow intention-to-treat principles using mixed models for repeated measures (MMRM).

**Discussion:**

The ACT-AD trial will assess the efficacy and safety of long-term, neuronavigated cerebellar iTBS in treating patients with mild-to-moderate AD.

**Trial registration:**

ClinicalTrials.gov, NCT06669182. Registered on 31 October 2024.

**Supplementary Information:**

The online version contains supplementary material available at https://doi.org/10.1186/s13063-026-10009-w.

## Introduction

AD is a progressive neurodegenerative disorder with a rapidly increasing prevalence that poses a global public health challenge [1]. The core pathological features of AD are the deposition of Amyloid-beta (Aβ) plaques and the formation of neurofibrillary tangles resulting from hyperphosphorylated tau protein, which trigger a cascade of synaptic dysfunction and neuronal loss, ultimately culminating in progressive cognitive decline [2]. Current therapeutic strategies are predominantly centered on pharmacological interventions, encompassing both symptomatic management and disease-modifying therapies. Nevertheless, even the most promising disease-modifying therapies face limitations such as inadequate blood–brain barrier penetration, side effects, high costs, and a narrow therapeutic window [3, 4]. Transcranial magnetic stimulation (TMS) is a non-invasive technique that employs electromagnetic induction to modulate cortical excitability and promote neuroplasticity, thereby restoring the dysfunctional brain networks associated with AD [5, 6]. However, the optimal stimulation parameters, precise target localization, and effective treatment protocols for TMS in AD have yet to be fully elucidated.

The cerebellum, which is involved in higher-order cognition and emotion, presents with less severe pathological changes in the early stage of AD compared to the cortex and hippocampus. This relative structural preservation makes it a promising target for neuromodulation [7]. The cerebellar Crus II region is a key node of interest within these cortico-cerebellar loops that is critically involved in cognitive processing [8]. This functional role is supported by its integration in a closed-loop circuit with prefrontal area 46 via its bidirectional connections [9], as well as by functional MRI evidence showing its activation during executive control, salience detection, and episodic memory/self-reflection through cortico-cerebellar loops [10]. Evidence suggests that cerebellar TMS may enhance multidomain cognitive function and functional connectivity in patients with AD [11, 12]. Our previous randomized, double-blind trial of neuronavigated iTBS targeting cerebellar Crus II region in AD patients found that cerebellar stimulation conferred significant cognitive benefits immediately post-treatment, but these effects waned by the 8-week follow-up [13]. This partial attenuation of benefit prompts the need for larger trials with extended treatment and maintenance protocols.

Therefore, we designed a multicenter, randomized, double-blind, sham-controlled trial to evaluate whether the intensive cerebellar iTBS protocol, followed by a maintenance phase, can effectively improve the functional outcome in patients with AD.

### Objectives

The purpose of this trial is to assess the efficacy and safety of an intensive course of cerebellar iTBS followed by a maintenance phase for improving the outcomes of AD.

### Trial design

**Fig. 1:**
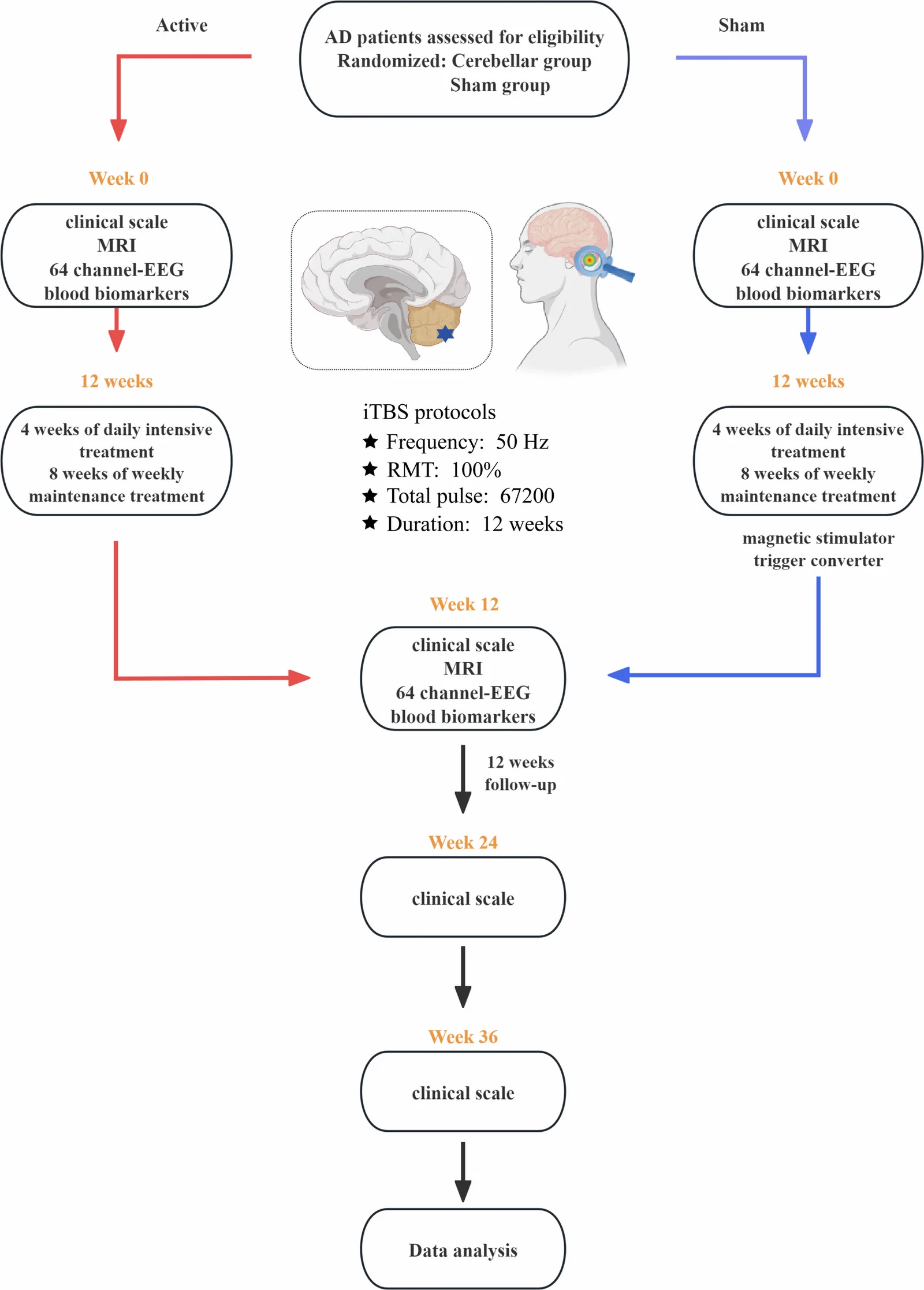
Study flowchart. AD, Alzheimer disease; MRI, magnetic resonance imaging; EEG, electroencephalogram; RMT, Resting motor threshold

**Fig. 2:**
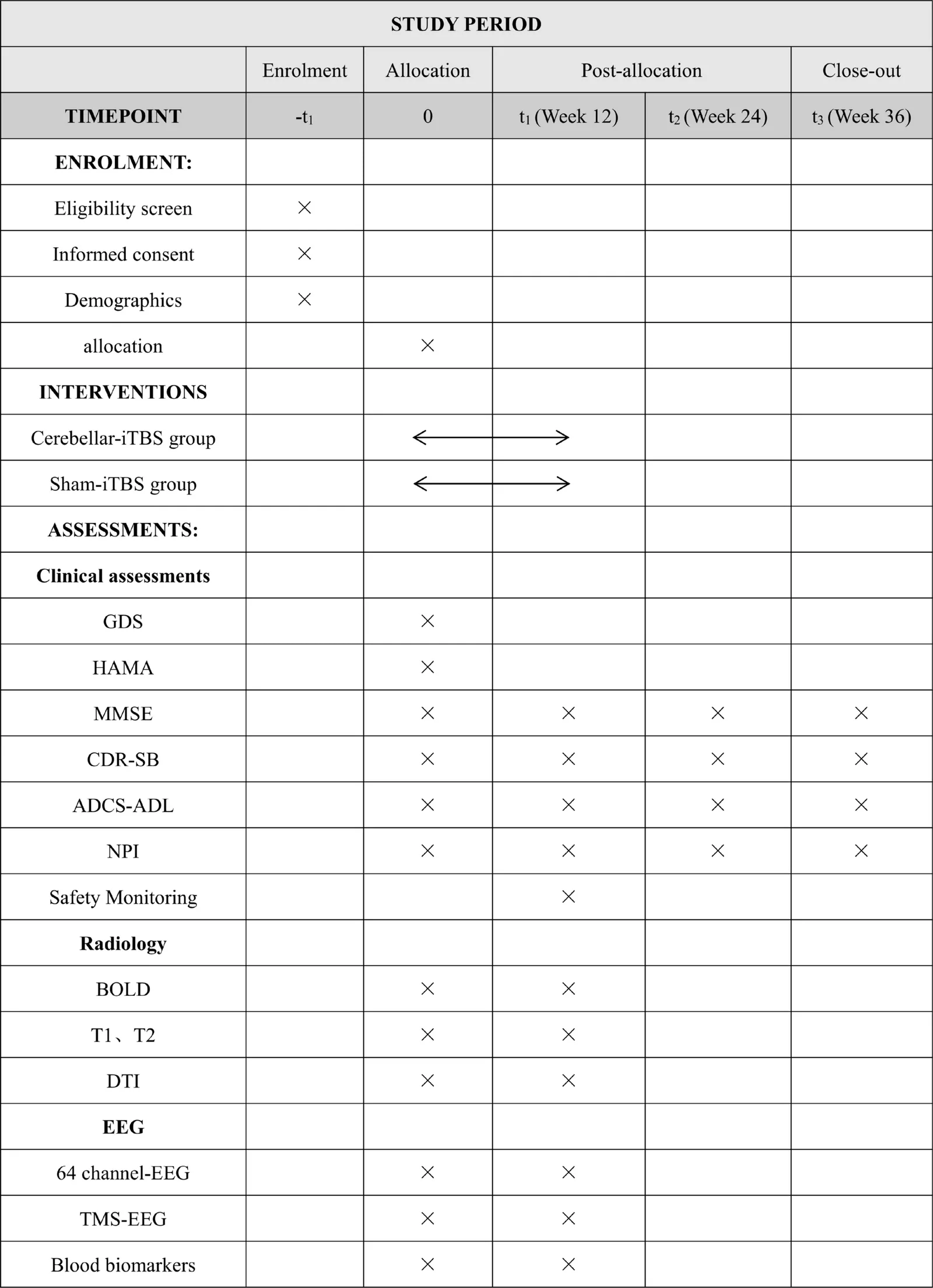
Schedule of patient enrollment, study interventions and outcome assessment. Abbreviations: ADAs-cog, Alzheimer’s Disease Assessment Scale-Cognition; ADCS-ADL, activities of daily living; BOLD, blood oxygenation level dependent; CDR-SB, Clinical Dementia Rating-Sum of Boxes; DTI, diffusion tensor imaging; EEG, electroencephalogram; GDS, Geriatric Depression Scale; HAMD, Hamilton Depression Rating Scale; HAMA, Hamilton Anxiety Rating Scale; MEP, motor evoked potential; MMSE, Mini-Mental State Examination; NPI, Neuropsychiatric Inventory; T1, T1-weighted image; T2, T2-weighted image; TMS, transcranial magnetic stimulation

The study is a multicenter, randomized, double-blind, sham-controlled trial. Patients will be randomly assigned to intervention group and control group Participants will receive 4 weeks of daily intensive treatment (Monday to Friday) followed by 8 weeks of weekly maintenance treatment. Follow-up assessments will then be conducted at week 24 and week 36 (Fig. 1). All the patients and researchers are blind to the group allocation, which was known only to the TMS operators. This study protocol adheres to the Standard Protocol Items: Recommendations for Interventional Trials (SPIRIT) guidelines. A SPIRIT figure (Fig. 2) outlines the schedule of enrolment, interventions, and assessments. The completed Standard Protocol Items: Recommendations for Interventional Trials (SPIRIT) checklist is provided as an additional file (see Additional file 1).

## Methods: participants, interventions, and outcomes

### Study setting

The patient recruitment will be conducted across four major hospitals in Shaanxi Province, China. The trial is coordinated by the Department of Neurology, Xijing Hospital (principal investigator: Wen Jiang, MD, PhD), with participation from three additional centers: the Department of Neurology, Tangdu Hospital (PI: Wei Zhang, MD, PhD), the Department of Neurology, Xi’an No. 3 Hospital (PI: Gejuan Zhang, MD), and the Department of Neurology, The First Affiliated Hospital of Xi'an Medical University (PI: Bei Zhang, MD, PhD). Data management, statistical planning, and analyses will be performed by the Department of Statistics of Fourth Military Medical University.

### Characteristics of the people who are needed for the trial

**Table:** 

Characteristic	The people we would expect to see included
Age	50 to 85 years old
Sex	We anticipate a distribution of biological sex (Male, Female) that is representative of the local AD population
Gender	We anticipate a distribution of gender identity (men, women) that is representative of the local AD population
Race, ethnicity and ancestry	All participants will be of Han Chinese ethnicity, recruited from Shaanxi Province, China
Socioeconomic status	Not a specific eligibility criterion
Geographic location	Participants will be recruited from urban areas in Shaanxi Province, China
Other characteristics relevant to the trial	Disease status: mild-to-moderate AD

### Eligibility criteria

Participants aged 50 to 85 years who meet the National Institute on Aging–Alzheimer's Association (NIA-AA) core clinical criteria for AD and have biomarker evidence of elevated phosphorylated tau (p-tau) and decreased Aβ42 will be recruited [14, 15]. Trained neurologists at every center will be requested to make a definite diagnosis of mild-to-moderate AD and confirm that each patient has met the inclusion criteria of the study. Written informed consent will be obtained from all patients or their guardians by the trained neurologists.

The inclusion criteria include (1) aged 50 to 85 years; (2) meet the core clinical criteria of NIA-AA for possible AD dementia, with confirmation of elevated p-tau and decreased Aβ (1–42) in Positron Emission Tomography (PET) or cerebrospinal fluid (CSF); (3) Mini-Mental State Examination (MMSE) score of 18–26 points, and a Clinical Dementia Rating (CDR) score of 0.5 or 1 points; (4) stable pharmacological regimen for AD (acetylcholinesterase inhibitors, NMDA receptor antagonists, or memantine therapy) for at least 12 weeks prior to enrollment; (5) has at least one adult caregiver; (6) written informed consent from patient or legal guardian.

Patients will be excluded if they have (1) neurodegenerative disorders other than AD; (2) significant intracranial focal or vascular pathology seen on brain MRI scan; (3) history of seizure (with the exception of febrile seizures in childhood); (4) major active psychiatric illness as defined by Diagnostic and Statistical Manual of Mental Disorders, Fourth Edition, Text Revision (DSM IV-TR) criteria, including major depressive disorder (current); schizophrenia; other psychotic disorders, bipolar disorder, or substance-related disorders (within the past 5 years); (5) Geriatric Depression Scale (GDS) score ≥ 8 points in baseline assessment; (6) cerebrovascular disease, severe infection, malignant tumor, or severe dysfunction of organs such as heart, liver, and kidney; (7) pregnancy or lactation; (8) contraindications for TMS or MRI, such as metal or implanted devices in the body (such as pacemakers, deep brain stimulators); and (9) participation in other AD-related trials within 6 months prior to enrollment.

### Who will take informed consent?

All eligible patients with mild-to-moderate AD and their guardians will be invited to participate. A trained clinical research coordinator will hold a face-to-face meeting to explain the study goals, cerebellar iTBS protocol, procedures, potential risks and benefits. During this meeting, participants will also be separately informed that blood samples and clinical data collected during the trial may be anonymized and stored for future research on AD. Informed consent must be obtained from both the patient and their guardian at least 24 h before the first iTBS session. Participants and guardians will be informed of their right to withdraw at any time without affecting standard medical care.

### Additional consent provisions for collection and use of participant data and biological specimens

The collection of biological specimens is part of this trial. Blood will be drawn before and after the intervention for the analysis of disease-relevant biomarkers. These samples may be anonymized and stored. Participants will be asked to consent separately to the long-term storage and future use of their biological samples. They can refuse this without any consequences for their main trial participation.

## Interventions

### Explanation for the choice of comparators

Patients will be randomly assigned to two different arms: (1) intervention group, receiving iTBS-TMS targeting on the cerebellar crus II at 100% of the resting motor threshold (RMT); (2) control group, receiving identical parameters of sham-TMS matched in auditory stimulation without the delivery of an electromagnetic pulse.

### iTBS treatment

This study employs a personalized, MRI-guided, dual-target rTMS protocol delivered with stereotactic neuromavigation for precision treatment. T1-weighted structural MRI is obtained from each participant before the initiation of rTMS. A frameless neural navigator system (Brain Science Tools BV, Utrecht, The Netherlands; www.neuralnavigator.com) will be used to ensure consistent coil targeting of the cerebellar. Three-dimensional images will be reconstructed based on individual pre-stimulation anatomical brain MRI scans. The intervention target is the cerebellar Crus II, determined by coordinates from the Montreal Neurological Institute (MNI) coordinate system (± 38, − 76, − 38). The final stimulation target coordinates will be confirmed on each subject's individualized brain structure using a neuronavigation system. The coil positioning procedure is as follows: first, the system is calibrated to the individual's head by registering anatomical fiducial points (nasion, tip of the nose, and bilateral tragus) to the MRI data. After initial registration, scalp sampling is performed to achieve precise surface matching. Following target identification, the appropriate TMS coil is selected and calibrated. Finally, the scalp projection of the target is precisely located under the guidance of the navigation system.

Stimulation will be performed using a Neuro-MS/D stimulator (Neurosoft, GTMEDI Technology Co., Ltd., Beijing, China) connected to a double-cone coil (external coil loop diameter, 125 mm; peak magnetic field, 4 Tesla). Prior to treatment (week 0), the RMT will be measured with the patient in a seated position. Using single-pulse TMS over the motor cortex representing the dominant hand, RMT is defined as the minimal intensity required to evoke observable thumb abduction in 5 of 10 consecutive stimulations [16]. The cerebellar iTBS will be administered at an intensity of 100% of RMT with the following parameters: a frequency of 50 Hz, with each burst comprising 600 pulses over 40 s. Each treatment session will consist of two such bursts delivered to the bilateral cerebellar Crus II, separated by an interval of 5 min, resulting in 1200 pulses per hemisphere and a total of 2400 pulses per session. The treatment includes a 4-week intensive phase with daily sessions (5 times per week), followed by an 8-week maintenance phase with weekly sessions. The final follow-up assessment will be conducted at week 24 and week 36.

### Sham-iTBS treatment

The sham-iTBS will be administered using parameters identical to cerebellar stimulation, with the magnetic stimulator trigger converted to ensure the coil delivers only auditory stimulation without electromagnetic energy output. In addition, a paired electrical stimulation device will be used to provide a mild scalp sensation via surface electrodes [17]. The TMS protocol is designed in full compliance with the safety guidelines by the International Federation for Clinical Neurophysiology [18].

### Training of investigators

To ensure the quality of the ACT-AD trial, all the investigators from every center are required to receive official training regarding the study protocol, MMSE, CDR-SB, Alzheimer’s Disease Cooperative Study–Activities of Daily Living (ADCS-ADL), Neuropsychiatric Inventory (NPI), GDS, Hamilton Depression Rating Scale (HAMD), Hamilton Anxiety Rating Scale (HAMA), as well as the clinical criteria of NIA-AA for possible AD dementia.

### Criteria for discontinuing or modifying allocated interventions

Participants may be discontinued from the study upon request, if lost to follow-up (at least five attempts have been made to contact the patient and their family members), or upon significant cognitive decline or emergence of TMS contraindications.

### Strategies to improve adherence to interventions

To minimize missing data and ensure protocol compliance, each participant and the site coordinator will receive a paper record card to track attendance at all scheduled TBS sessions. Participants and the site coordinator are instructed to mark a check after completing each treatment session. In case of any missed appointment, the site coordinator at each participating center will initiate telephone follow-up within 24 h to inquire about the reason for absence and address any potential issues.

### Relevant concomitant care permitted or prohibited during the trial

Participants must not receive any other form of brain stimulation therapy or initiate new cognitive training programs during the study. Established medications for AD are allowed if the dosage has been stable. All concomitant treatments will be documented.

### Outcomes

#### Primary outcome

The primary outcome is the change of CDR-SB core from baseline to week 12 between the cerebellar-iTBS group and sham-iTBS group.

#### Secondary outcomes

Secondary outcomes include changes in MMSE, ADCS-ADL, and NPI scores from baseline to Week 12, Week 24 and Week 36, as well as changes in CDR-SB at Week 24 and Week 36.

#### Exploratory outcomes

The exploratory outcomes include the changes in resting-state EEG characteristics and TMS-EEG measures from baseline to week 12. The changes of the plasma levels of P-tau 217, P-tau 181, Aβ1–42, Aβ1–40, Aβ1–42/Aβ1–40, NFL, and GFAP from baseline to week 12, The associations between these biomarker changes and clinical treatment outcomes. MRI metrics included changes from baseline to Week 12 in cerebellar–cortical functional connectivity, the Diffusion Tensor Image Analysis Along the Perivascular Space (DTI-ALPS) index, Default mode network (DMN)-related resting-state functional connectivity, cerebellar Amplitude of Low-Frequency Fluctuations (ALFF), Blood Oxygen Level-Dependent – Cerebrospinal Fluid (BOLD-CSF) coupling, and Choroid Plexus / Intracranial Volume(CP/ICV).

### Participant timeline

An overview of the trial design can be found in the flowchart (Fig. 1). The enrollment, intervention schedule, and outcome measures are illustrated in the SPIRIT diagram (Fig. 2).

### Sample size

The sample size was initially calculated based on 8-week pilot data, which showed a mean between-group difference of 0.40 points in CDR-SB change from baseline (pooled SD = 0.58) [13]. Using a two-sided alpha of 0.05 and 90% power for a superiority test at Week 12, the theoretical sample size was 90. Assuming an estimated 10% dropout rate, the final sample size was increased to 100, with 1:1 randomization.

### Recruitment

Participants for this study were recruited from the Department of Neurology across all participating centers. Our recruitment information was published on the official WeChat public platforms of each respective hospital. Potential subjects who met the inclusion criteria were then formally invited to enroll in the study.

## Assignment of interventions: allocation

### Randomization

To ensure allocation concealment, an independent statistician who is not involved in the trial will generate the allocation sequence using a computer-generated random number list. A stratified block randomization with fixed block sizes will be used, stratified by study center and age. After a participant is enrolled by the site investigators, the baseline data will be entered into the centralized, secure online randomization system (www.epiedc.com/kf.php) by a designated, independent researcher at each center. The system will then assign the participant to an intervention group in a 1:1 ratio. This process ensures that the sequence is concealed until the moment of assignment.

### Blinding

All of the participants, care providers, outcome assessors, and data analysts will be blinded to group allocation throughout the trial. Only the TMS operators will be unblinded. Patients will wear noise-canceling headphones to minimize auditory disturbance from TMS, and both intervention types will be delivered in identical settings. After treatment (week 12), patients will be asked to guess their group allocation to test the blinding integrity. For serious adverse events requiring knowledge of the treatment allocation for clinical management, authorized site personnel may initiate unblinding via the randomization system website. The system automatically documents the timestamp, user identity, and allocation revealed, and all such events must be reported to the clinical trial management center within 24 h.

### Data collection plan

#### Baseline and outcome data

At baseline, demographics data of gender, age, years of education, height, weight, family history, and time of onset will be collected. Clinical assessments will be evaluated using the MMSE, CDR, HAMA, HAMD and GDS. Those who meet the inclusion criteria and provide written informed consent will receive further evaluations of cognitive function, self-care ability, and neuropsychiatric status. A comprehensive battery including the MMSE, CDR, ADCS-ADL, and NPI will be administered at three time points: pre-treatment (week 0), post-treatment (week 12), and follow-up (week 24 and week 36).

The adverse events (AEs) associated with cerebellar iTBS will be monitored using a standardized form. Following each session, patients will be assessed for the presence and severity of symptoms, including headache, weakness, dizziness, eye/nose discomfort, jaw/teeth or facial discomfort, drowsiness, as well as other symptoms.

Multimodal data including structural/functional MRI (T1, T2, Blood oxygenation level dependent [BOLD], DTI), EEG (64-channel cap), and venous blood samples will be collected at Weeks 0 and 12 at the Xijing Hospital center for analysis of AD-related plasma biomarkers (P-tau 217, P-tau 181, Aβ1–42, Aβ1–40, Aβ1–42/Aβ1–40) (Fig. 2).

#### Plans for participant retention

To maximize participant retention and complete follow-up, several strategies will be implemented: (1) scheduling visits with flexibility to accommodate participants’ needs; (2) sending automated reminders via WeChat or phone call prior to each visit. For participants who discontinue the intervention, efforts will be made to collect at least the primary outcome and safety data at their scheduled time points.

#### Data management

The following procedures will be implemented to minimize bias and ensure data quality and protocol adherence: (1) a centralized training session will be conducted for all research coordinators prior to study initiation; (2) at each site, the principal investigator (PI) will oversee trial conduct and data quality; (3) monitoring visits will be conducted after every 5 patients enrolled per site to verify eligibility, informed consent, serious adverse events (SAEs), case report form (CRF) and data anomalies; (4) the Quality Control and Assurance Committee (QCAC) will generate monthly reports on recruitment, randomization, data completeness, and protocol adherence. Original study documents, including informed consent forms, CRF, and adverse event reports, along with de-identified MRI data in Digital Imaging and Communications in Medicine (DICOM) format, will be securely stored in locked cabinets within access-controlled offices. Blood samples for biomarker analysis will be archived in a dedicated − 80 °C freezer. All electronic data will be maintained on a secure computer protected by passwords and a firewall.

### Statistical analysis

In this study, patients will be analyzed according to the principle of intention-to-treat (ITT), with a planned total sample size of 100 participants (50 per group). Continuous and categorical variables will be summarized as mean (± SD)/median (IQR) and counts (percentages), respectively. Baseline characteristics will be summarized descriptively by treatment group without formal hypothesis testing. To compare the effects of cerebellar-iTBS on clinical symptoms between groups over time, the primary outcome, change in CDR-SB from baseline to Week 12, will be analyzed using a mixed model for repeated measures (MMRM), including treatment group, visit, treatment-by-visit interaction, and center as fixed effects; baseline CDR-SB, age, sex, and years of education as covariates. MRI and EEG data will be analyzed using ANOVA, with multiple comparison corrections. Missing data in the primary analysis will be handled using MMRM under the Missing-At-Random (MAR) assumption, using all available post-baseline data; multiple imputation under MAR and Missing Not At Random (MNAR) assumptions and complete-case analysis will be conducted as sensitivity analyses. Two-sided *p*-values < 0.05 in all tests will be considered significant. Statistical analyses will be carried out using SPSS (version 23), R (version 4.3.0), MATLAB and FSL.

### Data monitoring: formal committee

The PI at each center will be responsible for quality control by ensuring that the trial is conducted in compliance with the protocol, applicable guidelines, and regulations. The QCAC will prepare monthly reports on trial progress, including recruitment, randomization, protocol adherence, AEs/SAEs, and data completeness. All participating sites will conduct phase meetings and monthly monitoring visits to verify informed consent, eligibility criteria, data anomalies, and reported SAEs.

### Data monitoring: interim analysis

Not applicable to the study. Data analysis will begin only after all participants have completed the study.

### Harms

The harms include the incidence of cerebellar iTBS intolerance, AEs and SAEs throughout the study. The AEs associated with TMS are generally mild and transient, such as headache, scalp discomfort, or dizziness. In contrast, SAEs, like seizures, are extremely rare when established safety guidelines are followed.

### Auditing

The trial will undergo independent audits by the QCAC, conducted monthly and upon completion of site recruitment. Auditors will maintain full access to all source documents and research records. Findings will be reported directly to the trial steering committee, with mandatory corrective actions implemented for all identified deficiencies.

### Research ethics approval

This study will be conducted based on the Good Clinical Practice guidelines and the Declaration of Helsinki. The protocol has been approved by the Ethics Committee of Xijing Hospital and registered at (NCT06669182).

### Protocol amendments

All protocol amendments must be approved by the sponsor and the ACT-AD Study Group. Upon receiving ethics committee approval, the modifications of the protocol will be communicated to all trial investigators and the trial registry. Participants will be informed of relevant changes if necessary.

### Confidentiality

To ensure confidentiality, all participant data will be de-identified using a unique study code. Access is restricted to authorized personnel, with paper records stored in locked cabinets and electronic data on secure, password-protected systems. Biological samples are labeled with codes and stored in a dedicated − 80 °C freezer. Only anonymized data will be used for publication or sharing.

### Ancillary and post trial care

Participants will receive prompt and free medical care for any trial-related harm, which will be managed according to national regulations and institutional ethics standards. While no special post-trial care is planned beyond standard follow-up, compensation may be provided in cases of serious injury as per applicable laws and hospital policies.

### Dissemination policy

The results will be disseminated to broad audience, including health professionals, patients, the general public, and policy makers, through publication in a high-impact, international scientific journal and by reporting a summary to the ClinicalTrials.gov registry. The sponsor and funder have no authority over the publication decision.

## Discussion

The present study is a multicenter, randomized, double-blind, sham-controlled trial designed to evaluate the efficacy and safety of cerebellar iTBS in patients with AD. We will test the hypothesis that intensive cerebellar iTBS protocol, followed by a maintenance phase can effectively improve cognitive function in patients with mild-to-moderate AD. This trial will provide novel evidence to support the use of cerebellar iTBS as a therapeutic intervention for individuals with AD.

Previous studies have demonstrated that TMS as a novel physical therapy for improving outcomes in patients with AD [19]. However, some clinical trials, including our pilot study, have observed a decline in therapeutic effects during long-term follow-up [11, 13, 20]. A potential mechanism underlying this phenomenon may involve the modulation of synaptic plasticity by eliciting long-term potentiation (LTP) or long-term depression. However, transient stimulation may predominantly induce early-phase LTP, an initial stage that is independent of new protein synthesis and gene expression [21, 22]. Consequently, the resulting synaptic modifications and clinical benefits are unstable, which may be a key factor in the therapeutic attenuation after treatment cessation [23, 24]. Based on previous trial demonstrating that long-term TMS intervention can significantly slow the progression of AD, this study has accordingly designed an integrated protocol comprising intensive and maintenance phases to achieve consolidated and sustained therapeutic effects [25, 26].

Recently, the cerebellum has emerged as a focus of significant interest in cognitive neuroscience for its role in cognition. Despite its compact volume, the cerebellum surface is more tightly folded than the cerebral cortex and establishes extensive connections with nearly 80% of the neocortex [27]. The cerebellar role during the processing of the diverse cognition functions is probably mediated by its extensive connections to the cortical and subcortical areas [28–30]. Furthermore, hyperactivity in the cerebellar Crus II region during the pre-dementia stage of AD is hypothesized to be a compensatory mechanism mitigating early symptoms [31]. Di Lorenzo and colleagues further demonstrated that cerebellar iTBS induces impaired long-term potentiation-like plasticity in AD patients, indicating that altered cerebro-cerebellar plasticity plays a critical role in the pathophysiology of AD [32]. Notably, the cerebellum is the last region affected in the AD pathological cascade [33], marking it as a relatively resistant “survivor” [34]. Based on these findings, we propose that the cerebellum is an ideal neuromodulatory target for rTMS to promote cognitive improvement.

We will employ a multimodal approach to systematically investigate the therapeutic efficacy and underlying mechanisms of cerebellar iTBS in treating AD. The study will focus particularly on how cerebellar stimulation modulates functional brain networks, including the default mode, salience, and central executive networks [35–39], which are known to exhibit early connectivity disruptions in AD-related amyloid pathology [38, 40, 41]. We hypothesize that cerebellar iTBS may normalize the aberrant activity within these networks via cerebello-cortical pathways, thereby improving brain function. To evaluate this mechanism, we will integrate fMRI to assess network connectivity, EEG to capture neural oscillations dynamics, and plasma biomarkers, thereby constructing a comprehensive evidence chain from neural circuits to molecular levels [42–44].

Some limitations of our research should also be acknowledged. First, cerebellar targeting was not guided by individual functional MRI or electric field modeling, which should be addressed in future studies to optimize personalized stimulation site selection. Second, the absence of dynamic monitoring through CSF biomarkers (Aβ 42/p-tau181) or Aβ-PET imaging makes it difficult to validate the correlation between peripheral blood biomarkers and central pathology. Third, the 12 weeks intervention period may be insufficient to fully evaluate the long-term effects of cerebellar iTBS on neurodegenerative pathology.

In conclusion, we designed a multicenter, randomized, controlled trial to evaluate the efficacy and safety of an intensive cerebellar iTBS protocol with a maintenance phase for patients with mild-to-moderate AD. The findings are expected to establish a novel therapeutic mode for AD, and provide high-quality evidence for the application of neuromodulation as a core component of physical therapy for AD.

## Trial status

Protocol version: Version 2.0, 31 October 2024. Recruitment and follow-up of patients is currently ongoing. This trial will end in the third quarter of 2026.

## Supplementary Information

Additional file 1: SPIRIT checklist.

## Data Availability

No datasets were generated or analysed during the current study.

## Abbreviations

Aβ: Amyloid-beta
AD: Alzheimer’s disease
ADCS-ADL: Alzheimer’s Disease Cooperative Study–Activities of Daily Living
AEs: Adverse events
ALFF: Amplitude of Low-Frequency Fluctuations
BOLD: Blood oxygenation level dependent
BOLD-CSF: Blood Oxygen Level-Dependent – Cerebrospinal Fluid
CDR: Clinical Dementia Rating
CDR-SB: Clinical Dementia Rating Scale Sum of Boxes
CP/ICV: Choroid Plexus / Intracranial Volume
CRF: Case report form
CSF: Cerebrospinal fluid
DICOM: Digital Imaging and Communications in Medicine
DMN: Default mode network
DSM IV-TR: Diagnostic and Statistical Manual of Mental Disorders, Fourth Edition, Text Revision
DTI: Diffusion tensor imaging
DTI-ALPS: Diffusion Tensor Image Analysis Along the Perivascular Space
EEG: Electroencephalogram
fMRI: Functional connectivity
GDS: Geriatric Depression Scale
GFAP: Glial fibrillary acidic protein
HAMA: Hamilton Anxiety Rating Scale
HAMD: Hamilton Depression Rating Scale
ICA: Independent component analysis
IEDs: Interictal epileptiform discharges
iTBS: intermittent theta burst stimulation
ITT: intention-to-treat
LTP: Long-term potentiation
MAR: Missing-At-Random
MMRM: Mixed models for repeated measures
MMSE: Mini-Mental State Examination
MNAR: Missing Not At Random
MNI: Montreal Neurological Institute
MRI: Magnetic resonance imaging
NFL: Neurofilament light chain
NIA-AA: National Institute on Aging–Alzheimer’s Association
NPI: Neuropsychiatric Inventory
PET: Positron Emission Tomography
PI: Principal investigator
p-tau: Phosphorylated tau
QCAC: Quality Control and Assurance Committee
RMT: Resting motor threshold
SAEs: Serious adverse events
SPIRIT: Standard Protocol Items: Recommendations for Interventional Trials
TMS: Transcranial magnetic stimulation
